# Long-term Potentiation Decay and Poor Long-lasting Memory Process in the Wild Rodents *Proechimys* from Brazil’s Amazon Rainforest

**DOI:** 10.3389/fnbeh.2018.00002

**Published:** 2018-01-23

**Authors:** Marcia J. Guimarães Marques, Selvin Z. Reyes-Garcia, José E. Marques-Carneiro, Leonardo B. Lopes-Silva, Monica L. Andersen, Esper A. Cavalheiro, Fulvio A. Scorza, Carla A. Scorza

**Affiliations:** ^1^Disciplina de Neurociência, Departamento de Neurologia e Neurocirurgia, Escola Paulista de Medicina, Universidade Federal de São Paulo, São Paulo, Brazil; ^2^Departamento de Ciencias Morfológicas, Facultad de Ciencias Médicas, Universidad Nacional Autónoma de Honduras, Tegucigalpa, Honduras; ^3^Université de Strasbourg—INSERM U-1114—Neuropsychologie Cognitive, Physiopathologie de la Schizophrénie, Strasbourg, France; ^4^Departamento de Farmacologia, Escola Paulista de Medicina, Universidade Federal de São Paulo, São Paulo, Brazil; ^5^Departamento de Psicobiologia, Escola Paulista de Medicina, Universidade Federal de São Paulo, São Paulo, Brazil

**Keywords:** memory, long-term potentiation, learning, anxiety, hippocampus, plus-maze discriminative avoidance task

## Abstract

*Proechimys* are small terrestrial rodents from Amazon rainforest. Each animal species is adapted to a specific environment in which the animal evolved therefore without comparative approaches unique characteristics of distinct species cannot be fully recognized. Laboratory rodents are exceedingly inbred strains dissociated from their native habitats and their fundamental ecological aspects are abstracted. Thus, the employment of exotic non-model species can be informative and complement conventional animal models. With the aim of promoting comparative studies between the exotic wildlife populations in the laboratory and traditional rodent model, we surveyed a type of synaptic plasticity intimately related to memory encoding in animals. Using theta-burst paradigm, *in vitro* long-term potentiation (LTP) in the CA1 subfield of hippocampal slices was assessed in the Amazon rodents *Proechimys* and Wistar rats. Memory, learning and anxiety were investigated through the plus-maze discriminative avoidance task (PM-DAT) and object recognition test. In PM-DAT, both animal species were submitted to two test sessions (3-h and 24-h) after the conditioning training. *Proechimys* exhibited higher anxiety-like behavior in the training session but during test sessions both species exhibited similar patterns of anxiety-related behavior. After 3-h of the training, *Proechimys* and Wistar spent significantly less time in the aversive enclosed arm than in the non-aversive arm. But, at 24-h after training, Wistar rats remained less time in the aversive closed arm in comparison with the non-aversive one, while *Proechimys* rodents spent the same amount of time in both enclosed arms. In the object recognition test, both species were evaluated at 24-h after the acquisition session and similar findings than those of the PM-DAT (24-h) were obtained, suggesting that long-term memory duration did not persist for 24-h in the Amazon rodent. Field excitatory post-synaptic potentials recordings revealed that LTP decays rapidly over time reaching basal levels at 90 min after theta-burst stimulation in *Proechimys*, contrasting to the stable LTP found in the Wistar rats which was observed throughout 3-h recording period. These findings suggest a link between the LTP decay and the lack of 24-h long-lasting memory process in *Proechimys*. Nevertheless, why early-phase LTP in *Proechimys* decays very rapidly remains to be elucidated.

## Introduction

*Proechimys* (Rodentia-Echimyidae) are small terrestrial rodents from Amazon rainforest that play a critical role in the forest dynamics acting as seed predators and seed dispersers (Rojas-Robles et al., 2012; Amaral et al., 2013). They are generalists regarding the occupation of the forest territory and their existence is very well predicted by variables that qualify young or devastated forested areas (Lambert and Adler, 2000). These spiny rats are important contributors in woodland maintenance and regeneration. In the last years, studies have shown that *Proechimys* rodents exhibit unique structural and functional brain characteristics (Fabene et al., 2001, 2004; Arida et al., 2005; Scorza et al., 2010, 2011). Brains, as well as all animal traits, are extremely distinct and adjusted to the habitats in which animals evolved and developed (Carlson, 2012). Laboratory rodents are exceedingly inbred strains dissociated from their native habitats for generations thus lacking genetic and behavior diversity (Klaus and Amrein, 2012; Keifer and Summers, 2016). Fundamental ecological aspects are entirely abstracted in these laboratory animals. In principle, every species can add something to the comprehension and progress of science (Manger et al., 2008). By taking account of similarities and differences among species, researches can appreciate unique features and general principles of the nervous system (Keifer and Summers, 2016). Therefore, wild non-model species can complement conventional animal models and have something to offer to our comprehension of the brain (Brenowitz and Zakon, 2015).

Memory endows animals with the ability to learn and adjust their behavior based on past experiences and is one of the most puzzling processes of the brain. Ramón y Cajal (1894) was the first to speculate that memory relies on strengthening of synapses between neuronal cells. In addition to propose the value of environmental complexity and its influences on animals (Hebb, 1947), generating interest in how the environment impacts both the brain and behavior, Hebb (1949) also inaugurated the ideas of activity-dependent synaptic plasticity and cell-assembly formation in an attempt to explain the processes of learning and memory (Mayford et al., 2012; Poo et al., 2016). In the hippocampal formation, the “Hebbian” mechanisms gained great attention after the description of long-term potentiation (LTP) by Bliss and Lømo (1973). They showed that a brief high-frequency stimulation at excitatory synapses in the brain led to long-lasting enhancement of synaptic transmission efficiency (Bliss and Lømo, 1973), a discovery that revealed the plasticity of synapses, an essential property of learning and memory (Lømo, 2016). Notwithstanding, LTP has been cautiously acknowledged in the initial articles as the physiological foundation of learning and memory (Bliss and Gardner-Medwin, 1973; Bliss and Lømo, 1973), it is still currently debated whether LTP is related to these brain processes, although investigations on animals deliver robust evidence that it is indeed necessary (Morris et al., 1986; McHugh et al., 1996; Frey and Morris, 1997; Whitlock et al., 2006; Shema et al., 2007; Cooke and Bear, 2010; Madroñal et al., 2010; Sacktor, 2011; Glanzman, 2013; Nabavi et al., 2014).

Taken together, this work was conceived with the goal of promoting comparative studies for memory process and synaptic plasticity between the exotic wildlife populations in the laboratory and traditional rodent model. We surveyed a type of synaptic plasticity intimately related to memory encoding in animals. Using theta-burst paradigm that imitates *in vivo* firing patterns of hippocampal neurons, *in vitro* LTP in the CA1 subfield of hippocampal slices was assessed in the Amazon rodents *Proechimys* and Wistar rats. Memory, learning and anxiety were investigated through the plus-maze discriminative avoidance task (PM-DAT) and object recognition test.

## Materials and Methods

### Animals

Male *Proechimys* rodents (*n* = 18), originally from the Amazon rainforest, were bred in a colony established at the Neuroscience Laboratory’s facility (Federal Register-IBAMA number 1561643) of Escola Paulista de Medicina/Universidade Federal de São Paulo (EPM/UNIFESP). Male Wistar (*n* = 18) rats were acquired from CEDEME/UNIFESP. Throughout the study, the animals were maintained at a constant temperature of 22 ± 1°C, 12-h light-dark cycle and with free access to food and water. All animal procedures were carried out in accordance with the Ethical and Practical Principles of the Use of Laboratory Animals with the approval of the Ethical Committee of UNIFESP (CEUA 8615110915). Precautions were taken to minimize the number of rodents utilized in the experiments.

### Plus-Maze Discriminative Avoidance Task (PM-DAT)

**Figure 1:**
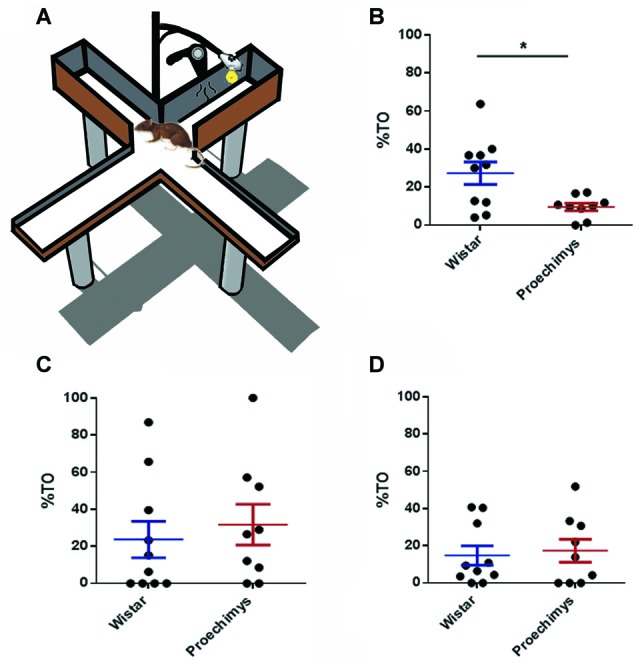
**(A)** Plus-maze discriminative avoidance task (PM-DAT). **(B)** Percent time (%TO) spent by Wistar and *Proechimys* in the open arms 10 min-exposure training session. **(C)** Three-minutes exposure test session after 3 h of the training session. **(D)** Three-minutes exposure test session after 24 h of the training session (*p < 0.05).

The PM-DAT is an useful test for investigating hippocampal-dependent memory involving emotionality and conveniently allows the simultaneous study of the learning, memory and anxiety (Silva and Frussa-Filho, 2000; Rachetti et al., 2013; Frussa-Filho et al., 2016; Leão et al., 2016b). The device used was a modified elevated plus-maze, 52 cm above the floor, composed of two closed arms (48 × 16 × 50 cm) opposite to two open arms (48 × 16 cm). Wistar rats (*n* = 10) and *Proechimys* rodents (*n* = 9) were conditioned to decide between the two enclosed arms (in one of them, a 100-W lamp and a 2200 W hair drier, as aversive stimuli, were both placed above the end of the arm) whilst avoiding the open arms of the device (Figure 1A). During the training session, which lasted 10 min, each rodent was positioned in the center of the device and each time the animal penetrated the aversive enclosed arm, the aversive stimulus was generated until the rodent left this arm. Then, the animals were submitted to two test sessions of 3 min each, which occurred 3 h and 24 h after the conditioning training session. In the test sessions, the rodents were put on device but aversive stimuli were not actioned, although the hair-dryer and the lamp stayed positioned. Rodents were randomly tested and, after each session, a 5% alcohol solution was used to clean the device. Learning was assessed by time the animal remained in the aversive enclosed arm during training session (Silva et al., 2004; Carvalho et al., 2006; Patti et al., 2006). In turn, memory was assessed by time spent in the aversive enclosed arm during the test sessions (Silva and Frussa-Filho, 2000; Rachetti et al., 2013; Leão et al., 2016b). Anxiety-like behavior was given by the percent time spent in the open arms (amount of time the animal remained in the open arms/total time spent in both open and closed arms) of the device (Silva et al., 2000; Calzavara et al., 2004; Leão et al., 2016a).

### Object Recognition Task

A rectangular acrylic box (66 × 40 × 30 cm) was used in the experiments. The objects to be recognized were made of glass and presented different shapes and textures, they were tested in advance and animals showed no preferential exploration. Exploration was established when rodents sniffed or touched the object with the nose and/or forepaws. Before the test, the Wistar (*n* = 8) and *Proechimys* (*n* = 9) were habituated to the experimental box by allowing them to freely explore it during 10-min every day for consecutive 5 days. No stimulus object was placed inside the arena during habituation. The rodents were individually inserted into the box with two distinct objects (A and B) and left to freely explore them for 5-min. Twenty four hours later, animals were placed again in the same experimental box for a 5-min, but now they were presented to the familiar object (A) and to a novel object (C).

### Brain Slice Preparation

Brain slice preparation was produced as described previously (Salar et al., 2016). Briefly, rodents were anesthetized with 1% isoflurane dissolved in 70% N_2_O and 30% O_2_. Then, adult *Proechimys* (*n* = 4) and Wistar rats (*n* = 4) were decapitated and brains quickly removed. Horizontal hippocampal slices were made in ice-cold artificial cerebrospinal fluid (aCSF) at 4 ± 0.5°C temperature containing (in mM): NaCl 129, NaHCO_3_ 21, KCl 3, CaCl_2_ 1.6, MgSO_4_ 1.8, NaH_2_PO_4_ 1.25, and glucose 10, saturated with 95% O_2_ and 5% CO_2_. Using a vibratome (LEICA VT 1200S), 400 μm thick brain slices were generated and promptly moved to an interface chamber perfused with aCSF at 36 ± 0.5°C (flow rate: 1.5–2 ml/min, pH 7.4, osmolarity: 295–300 mOsmol/L) where they were allowed to recover for 2–3 h.

### *In Vitro* Electrophysiology—Stimulation and Recording

Extracellular field potentials (FPs) were recorded from the* stratum radiatum* of cornu *Ammonis* 1 (CA1) area. The recording electrode consisted of a chlorinated silver wire inserted into a glass pipette filled with 154 mM NaCl (5–10 MΩ). Schaffer collateral axons received the orthodromic stimuli through a bipolar twisted electrode (Ni-Cr wire with 50 μm diameter tip) positioned in the *stratum radiatum* of the CA1 hippocampal subfield. All data were low-pass filtered at 3 kHz, digitized at 10 KHz and stored on computer disk using a CED 1401 interface for off-line analysis using Spike 2 v6.09 (CED-1401, Cambridge, UK). Before starting the LTP protocol, input-output (I/O) relationships of stimulus intensity vs. field excitatory postsynaptic potentials (fEPSP) magnitude were performed in order to determine the maximal response after constant increments of stimulus intensity until no further increase in the fEPSP amplitude. From that, stimulus intensities were adapted to generate a fEPSP slope of 50%–60% of the maximum obtained from the I/O. Field EPSP slope values were accessed after the fiber volley in order to avoid the influence of other sources of current flow. Twenty minutes of stable baseline recording period was established before application of the high frequency stimulation, in which paired pulses were delivered with an inter-stimulus interval of 50 ms. LTP was induced by Theta Burst Stimulation (TBS) protocol (Liu et al., 2016), which consists in 10 bursts repeated at 200 ms intervals, with four pulses at 100 Hz for each burst, at the same stimulus intensity as the baseline, delivered to the Schaffer collaterals and the responses were recorded during 180 min.

### Statistical Analysis

Electrophysiological data were analyzed using custom-made scripts (© Jan-Oliver Hollnagel, MATLAB R2013b; Salar et al., 2016). The fEPSP slope was measured between 20%–80% of its maximal amplitude. In order to verify the potentiation after TBS, field EPSP slopes were normalized relative to the averaged baseline response. Statistical analysis of behavioral and electrophysiological data was reported as mean ± standard error of the mean (SEM). Shapiro–Wilk test was used to determine normality. On behavioral tests, the statistical significance was assessed by non-parametric Mann-Whitney test, since less than 30 animals were used in the study, therefore the sample is not reliable to perform parametric approach (Ghasemi and Zahediasl, 2012). Statistical analysis for LTP was performed using two-way ANOVA (fEPSP slope at different times) followed by Bonferroni *post hoc* test. *p* < 0.05 (*) was considered to indicate a significant difference. Statistical analysis performed using Prism 5.00 (GraphPad Software, Inc., San Diego, CA, USA).

## Results

### Plus-Maze Discriminative Avoidance Task (PM-DAT)

The wild Amazon rodents exhibited high anxiety-like behavior in the training session. The Wistar group spent more time on the open arms of the equipment than the *Proechimys* group (*U* = 18.00; *p* < 0.05; Figure 1B), throughout the training session. However, during test sessions, both species of animals exhibited similar patterns of anxiety-related behavior. No differences, between Wistar e *Proechimys*, were observed in the amount of time spent in the open arms of the device during the test sessions that occurred 3 h (*U* = 36.00; *p* > 0.05) and 24 h (*U* = 45.00; *p* > 0.05) after the training session (Figures 1C,D).

**Figure 2:**
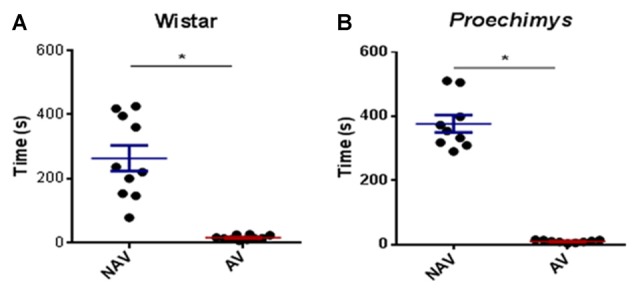
Training session in the plus-maze discriminative avoidance. Time spent by Wistar **(A)** and *Proechimys* **(B)** in aversive (AV) and non-aversive (NAV) enclosedarms (*p < 0.05).

In the training session both species of rodents spent less time in the aversive closed arm in comparison to the amount of time spent in the non-aversive closed arm. Wistar (*U* = 0.00; *p* > 0.0001) and *Proechimys* (*U* = 0.00; *p* > 0.0001; Figures 2A,B).

**Figure 3:**
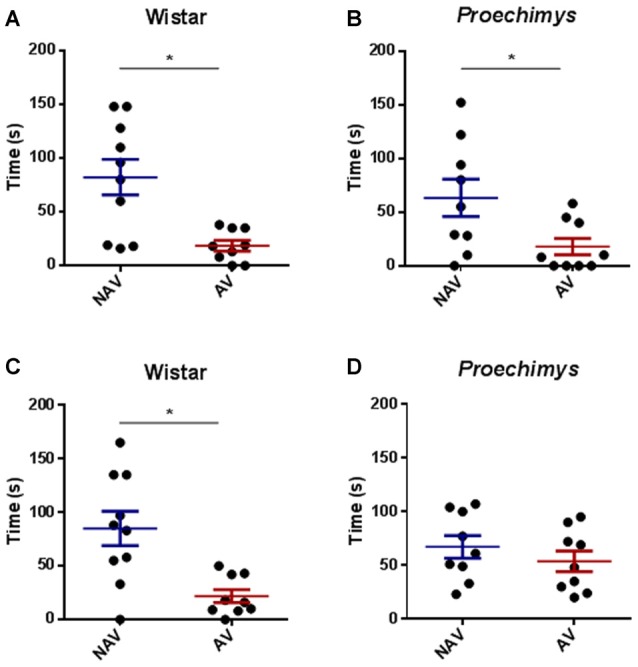
Test session in the plus-maze discriminative avoidance. Test session after 3 h of training session, time spent by Wistar **(A)** and *Proechimys* **(B)** in aversive (AV) and non-aversive (NAV) enclosed arms. Test session after 24 h of the training session, time spent by Wistar **(C)** and *Proechimys* **(D)** in aversive (AV) and non-aversive (NAV) enclosed arms (*p < 0.05).

In the test session, after 3 h of the training, both groups spent significantly less time in the aversive enclosed arm when compared to the time spent in the non-aversive enclosed arm. Wistar (*U* = 23.00; *p* < 0.05) and *Proechimys* (*U* = 17.50; *p* < 0.05; Figures 3A,B), suggesting that both animal species remembered the aversive situation.

In the test session, 24 h after training, Wistar rats remained less time in the aversive closed arm in comparison with the non-aversive arm (*U* = 21.50; *p* < 0.05; Figure 3C), although *Proechimys* rodents exhibited no difference in the amount of time spent in both enclosed arms (*U* = 19.00; *p* > 0.05; Figure 3D), suggesting that long term memory duration did not persist for 24 h in *Proechimys*.

### Object Recognition Task

**Figure 4:**
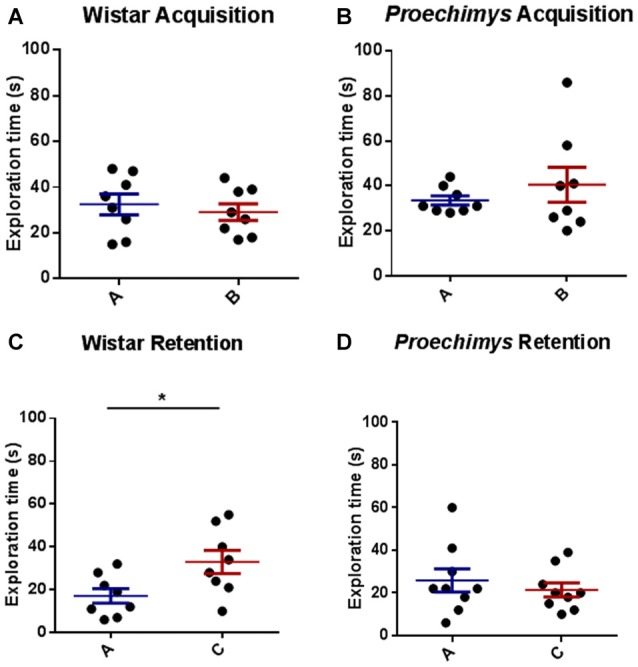
Object recognition task. Acquisition trial, exploration time spent by Wistar **(A)** and *Proechimys* **(B)** in object A and B. Retention trial, exploration time spent by Wistar **(C)** and *Proechimys* **(D)** in familiar and novel object (*p < 0.05).

The comparison between the time spent to explore both objects (A and B) revealed an equivalent exploration time between the different groups of rodents in the acquisition trial, Wistar (*U* = 27.50 and *p* > 0.05) and *Proechimys* (*U* = 31.50 and *p* > 0.05; Figures 4A,B). Wistar rats, 24 h later of the acquisition phase, spent significantly more time exploring the novel object compared with the familiar object (*U* = 12.50 and *p* < 0.05; Figure 4C). In contrast there was no significant difference in exploration of the novel object when compared to familiar object in *Proechimys* rodents (*U* = 33.00 and *p* > 0.05; Figure 4D), suggesting that long term memory duration did not persist for 24 h in *Proechimys*.

### Long-term Potentiation

**Figure 5:**
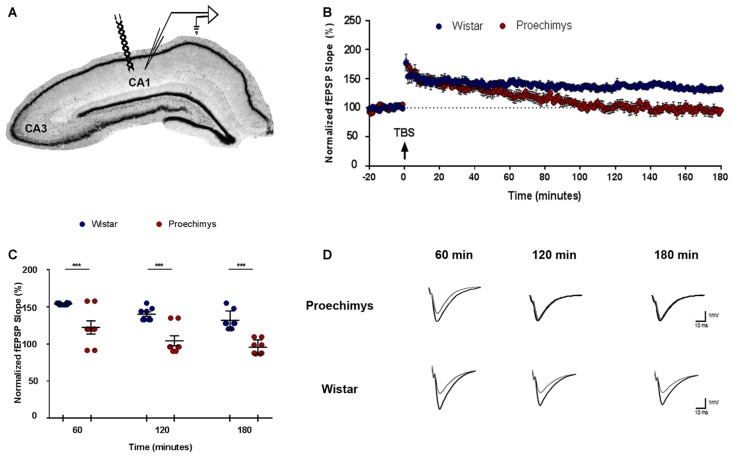
Long-term potentiation (LTP) in *Proechimys* and Wistar rodents. **(A)** Illustration shows the position of both electrodes recording and stimulation placed in CA1 hippocampal subfield. **(B)** Time evolution of fEPSP slopes of *Proechimys* and Wistar. Arrow indicates when theta burst stimulation (TBS) protocol was delivered. **(C)** The graph represents the average of fEPSP slope at 60, 120 and 180 min after TBS. **(D)** Representative superimposed recordings of averaged fEPSP before (gray trace) and 60, 120 and 180 min after (black trace) TBS in *Proechimys* and Wistar (***p < 0.05).

In order to evaluate LTP under physiological condition, animals that underwent behavioral tests were not used. Eight slices per animal group (*n* = 16) were used in the experiments (Figure 5A). There was no significant effect of interaction between groups (*F*_(2,42)_ = 0.5335; *p* > 0.05). Bonferroni’s *post hoc* test showed that the potentiation in the slope of fEPSP was significantly different (*F*_(1,42)_ = 64.84; *p* < 0.0001) between, *Proechimys* (17.2 ± 8.4%) and Wistar (54.4 ± 1.2%) at 60 min after TBS (Figure 5C). In *Proechimys*, fEPSP slope potentiation decayed over time reaching basal levels at 90 min after TBS, contrasting to Wistar rats (10.1 ± 7.1% vs. 40.1 ± 4.1%, respectively) in which potentiation was stable during recordings (e.g., fEPSP slope potentiation at 180 min after TBS, *Proechimys* 1.5 ± 3.1%, Wistar 33.3 ± 6.0%; Figures 5B,D). The results revealed differences associated with the maintenance phase of LTP between *Proechimys* and Wistar, since LTP was successfully induced in both animal species after TBS protocol.

## Discussion

In the PM-DAT we aimed to concomitantly assess learning, memory and anxiety (Silva and Frussa-Filho, 2000). At 3 h following training session, the animals returned to the apparatus for a memory retention test and both *Proechimys* and Wistar rodents were successful in remembering the task. However, at 24 h after training session, divergent long-term memory abilities were found and only Wistar rats exhibited memory retention. The results obtained in Wistar rats are in accordance with similar studies in the literature (Silva et al., 2000; Rachetti et al., 2013) but this is the first investigation of memory in the *Proechimys* rodents.

Studies using the PM-DAT have suggested that the 3-min test session, in comparison to the 10-min training session, is not long enough for animals to learn that although aversive devices are still present in the apparatus, they are not activated (Silva et al., 2000; Frussa-Filho et al., 2016). However, it could be different for *Proechimys*, since previous studies were performed in Wistar rats and PM-DAT was not validated for these wild rodents. Hence, through the object recognition test, one of the most commonly used behavioral tests in rodents (Antunes and Biala, 2012; Leger et al., 2013), 24-h long-term memory of *Proechimys* was assessed and the same results as those of PM-DAT were found. The findings obtained using Wistar rats are in accordance with previous studies (da Silveira et al., 2013; Furini et al., 2015).

The ability to remember and learn is absolutely fundamental for survival and every animal has its own physiological attribute, so one cannot truly compare the memory of two different species. Additionally, one can argue that under standard laboratory housing conditions, animals are exposed to potentially stressful and anxiogenic environmental challenges (Morgan and Tromborg, 2007) which may unfavorably impact the behavioral assessments, especially in the case of a wild-derived animal such as *Proechimys*. However, that does not seem to be the case here because higher anxiety-like behavior exhibited by *Proechimys’s* during one trial training session, when animals could be affected by the novelty*-*induced stress (File et al., 1990; Schneider et al., 2011), disappeared when they were tested at 3 h and 24 h after training. These findings obtained through PM-DAT suggested that *Proechimys* were not prone to higher anxiety levels than Wistar rats. Nevertheless, it is not possible exclude that the higher level of stress in *Proechimys* during acquisition task may have affected long-term maintenance of memory. Similarly, it is unlikely that higher levels of anxiety could explain the poor performance of *Proechimys* rodents in the novel object recognition test since both species showed similar total exploration time in both acquisition (when an object is introduced for the first time) and training sessions. The 5% alcohol solution is perhaps a limitation of our study since it may be not sufficient to avoid olfactory cues in the case of interspecies experiments. However, we cannot totally rule out that poor performance could be related to novelty avoidance due to higher emotionality.

Thereby, learning and memory are required for all animals to adapt and survive in their environment. Recent investigations fulfill several of the criteria that are required and sufficient to link learning/memory and LTP of synaptic strength between hippocampal neurons (Pastalkova et al., 2006; Whitlock et al., 2006). Current evidence has showed a possible relation between the maintenance of memory and the maintenance of LTP (Pastalkova et al., 2006; Sacktor, 2008).

Using TBS, here we found that LTP was induced in both *Proechimys* and Wistar rats but LTP of the excitatory postsynaptic potentials of the CA1 cells of *Proechimys* failed to be maintained over the course of 180 min of recording. *Proechimys*’s LTP potentiation decayed over time reaching baseline levels at 90 min after TBS, in contrast to Wistar rats in which potentiation could be observed throughout 3-h recording period. As shown in Figure 5C, fEPSP recorded in *Proechimys*’s brain slices showed that TBS could only produce a rapid decaying LTP. The literature reports that even weak protocols of induction can usually induce LTP that can last several hours in rodents (Lu et al., 2011; Dong et al., 2015). Theta-burst and tetanic stimulation have been the most frequently used experimental paradigms to induce LTP (Cao and Harris, 2014). Maybe, theta stimulation used in our work could be a very weak stimulation for *Proechimys*. For this reason, we also assessed LTP using different tetanic stimulation protocols, even multiple tetani in quick succession, but with similar results as those found with TBS (data not shown). Nevertheless, why early-phase LTP in *Proechimys* decays very rapidly remains an open question. It will be necessary to examine the adaptive function of cognitive processes in *Proechimys* thus allowing to predict what properties this cognitive system needs to have. Still, *in vivo* studies in *Proechimys* addressing the mechanisms underlying LTP maintenance and how this process is linked to memory are necessary.

## Author Contributions

MJGM, JEM-C and LBL-S: performed and analyzed the behavioral experiments. SZR-G: performed and analyzed the *in vitro* electrophysiology experiments. MLA and EAC: critical revision of the manuscript. MJGM: interpretation and wrote the manuscript. FAS: design the work and data analysis interpretation. CAS: conception and design the work, perform data analysis, interpretation and wrote the manuscript.

## Conflict of Interest Statement

The authors declare that the research was conducted in the absence of any commercial or financial relationships that could be construed as a potential conflict of interest.
